# Test Rig for the In Situ Measurement of the Elastic Tooth Deflection of Plastic Gears

**DOI:** 10.3390/polym15071732

**Published:** 2023-03-30

**Authors:** Christoph Herzog, Dietmar Drummer

**Affiliations:** Institute of Polymer Technology (LKT), Friedrich-Alexander Universität Erlangen-Nürnberg, 91054 Erlangen, Germany

**Keywords:** elastic tooth deflection, plastic gears, in situ gear testing

## Abstract

A new steel–plastic gear set testing methodology has been developed at the Institute of Polymer Technology (LKT). The in situ gear test rig analyses the timing differences between the index pulses of rotary encoders on the input and output shaft. This measurement principle enables the continuous measurement of the elastic tooth deflection on the one hand and permanent deformations and wear on the other hand by switching between a high loading torque and a low measuring torque. However, the elastic tooth deflection measurement using this principle has not yet been validated. Therefore, in situ gear tests using polybutylene terephthalate (PBT) gears were performed to evaluate the elastic tooth deflection of the plastic gear during operation. The results were compared to the results of pulsator tests. The comparison shows a very good correlation between the results of the newly developed in situ gear test rig and the well-established pulsator test rig. However, it has been shown that the test rig design creates a measuring offset due to angular displacements of the shafts due to torsion of test rig components.

## 1. Introduction

Thermoplastic gears can be found in nearly any kind of power transmission system. Due to their beneficial properties, they are widely used in various industrial sectors such as automotive, medical engineering and electronics [1]. Compared to metal gears, they show numerous advantages such as economic production by injection molding [1], dry run capability [2], lightweight construction [3], possibility of material modification [2] and noise reduction [4]. In terms of achievable lifetime and loading capacity, steel–plastic gear sets are the ideal pairing [2].

One major difference between plastic and metal gears is the viscoelastic material behavior of thermoplastics [2] which leads to pronounced tooth deflection during operation. The elastic tooth deflection influences the load sharing of the plastic gear’s teeth and therefore determines the resulting stresses [5]. Thus, detailed knowledge about the elastic tooth deflection during operation is essential for the lifetime-oriented design of steel–plastic gear sets.

Nevertheless, until now, there have been no experimental studies covering the elastic tooth deflection of plastic gears during operation. Therefore, this contribution presents the validation of a new gear testing methodology developed at the Institute of Polymer Technology (LKT) which enables the continuous measurement of the elastic tooth deflection during the test run. Experimental data of the elastic tooth deflection can further be used to optimize contact simulation models for calculating the stress concentrations of the plastic gear’s teeth.

### 1.1. Elastic Tooth Deflection of Plastic Gears

As far as the elastic tooth bending of plastic gears is concerned, different analytical and simulative approaches exist in the literature. However, there is considerably less research on experimental approaches.

According to VDI 2736 [6], the low elastic modulus of plastic gears leads to tooth deformations which act as pitch deviations. The deformation of the tooth tip in the circumferential direction λ can be approximately calculated according the following equation [6]:

(1) $$\lambda =\frac{{7.5\cdot F}_{t}}{b\cdot \cos\beta }\cdot \left(\frac{1}{E_{1}}+\frac{1}{E_{2}}\right)$$

with the nominal tangential force being $F_{t}$ the face width b, the helix angle β and the elastic modulus of the pinion ($E_{1}$) and gear ($E_{2}$) [6].

Walton and Shi [7] investigated the discrepancies between different analytical rating procedures for the tooth root bending stresses of plastic gears. These rating procedures are all based on the Lewis equation, which describes the strength of a gear tooth as follows [8]:

(2) $$W_{s}=s\cdot p\cdot F\cdot y$$

with the safe bending load of the gear tooth being $W_{s}$, the safe working stress of the material s, the circular pitch of the gear p, the face width of the gears F and the tooth form factor y.

However, when the tooth temperature rises due to friction during operation, the elastic modulus of the plastic gear decreases. Consequently, the tooth geometry changes and the bending stresses decrease. The rating procedures investigated by Walton and Shi [7] contain different correction factors for instance for lubrication, humidity and temperature. This leads to wide discrepancies between the results of the different approaches. In addition, the lack of experimental data makes the evaluation of these rating procedures difficult. In summary, it can be said that analytic calculations can only partly and simply describe the actual operating behavior.

Van Melick [5] studied the influence of tooth bending on the kinematics and stresses of gear pairs by performing finite element analyses. The results show that due to changes in load sharing, the bending stresses are reduced to roughly 2/3 of the theoretical root stress because of elastic tooth deflection. However, the elastic tooth bending leads to more severe loading at the end of the meshing cycle and to an extension of the contact line perpendicular to the theoretical contact line. A decreasing elastic modulus leads to lower stresses around the pitch point, but high stress peaks occur because the extended contact path tip of one gear comes into contact with the other gear. The consequences are unfavorable pressure–velocity combinations (PV value). The PV value can be up to seven times higher than the theoretical value.

Terashima, Tsukamoto and Shi [9] present an experimental approach for measuring the tooth deflection of plastic gears and the corresponding rotational delay of the steel gear. The tooth deflection is measured by evaluating the angular displacement between a steel and plastic gear under load. The main conclusions are that the plastic gear’s tooth deflection causes 98% of the total deflection of the two meshing teeth resulting in a rotational delay of the steel gear. As a consequence, severe wear occurs in the dedendum area of the plastic gear’s teeth.

Nevertheless, the experimental setup used by Terashima, Tsukamoto and Shi [9] does not exactly reproduce the gear meshing during operation. Therefore, influences of progressing tooth flank wear and changes in temperature on the tooth deflection remain unconsidered.

### 1.2. Current Methods and Designs for In Situ Gear Testing

Two main categories for in situ gear test rigs can be distinguished: test rigs for condition monitoring and test rigs for wear and deflection measurement. Condition monitoring aims for the efficient planning of maintenance and mitigation of downtime due to failure of machine elements [10]. These test rigs use sensor data to predict failure mechanisms and remaining lifetime [11]. Numerous studies focus on the detection of acoustic and vibrational signals as an expression of typical types of gear failure such as cracks [12,13], tooth flank wear [12,14] and pitting [12,15]. Other sensors, such as oil wear debris sensors [10] and thermographic cameras [16], are less commonly used.

Since condition monitoring test rigs typically do not directly quantify the underlying process of gear failure, such as tooth flank wear, there are in situ gear test rigs particularly designed for wear and deflection measurement. For the continuous tooth flank wear measurement only a few concepts are described in the literature.

Sosa et al. [17] use a tactile profile measurement sensor for analyzing the tooth contour in situ. Limitations of this approach are that the test run has to be stopped for measuring and the plastic gears have to be big enough for the measuring tip to reach the relevant areas of the tooth flank. The concept of Yousef et al. [18] is based on the gravimetric measurement of the plastic gear, assuming that the gear weight decreases proportionally to the gear wear. However, this approach is only applicable if the wear debris completely leaves the system. The experimental setup of Hooke et al. [19] consists of a gear pairing, which is mounted on a rotatable block. The block is loaded with a weight on a loading arm. The construction is balanced during operation but rotates downwards with progressing wear. The main limitation of this concept is that wear and elastic deformation cannot be measured separately.

Optical gear measurements enable a more comprehensive and detailed analysis of the operating behavior of plastic gears during the test run. Tanaka et al. [20] analyzed pitting of metal gears using the scattering of a laser beam. However, these optical approaches cannot measure the tooth flank in contact optically. Therefore, the elastic deformation of polymer gears during the test run cannot be detected. Černe, and Petkovšek [21] used high-speed camera images and digital image correlation to analyze tooth deflection during operation. Despite these approaches being promising, the quality of the results depends on the rotational speed. At high speeds, the measurement accuracy might not be sufficient anymore.

### 1.3. Functional Principle of the LKT In Situ Gear Test Rig

**Figure 1:**
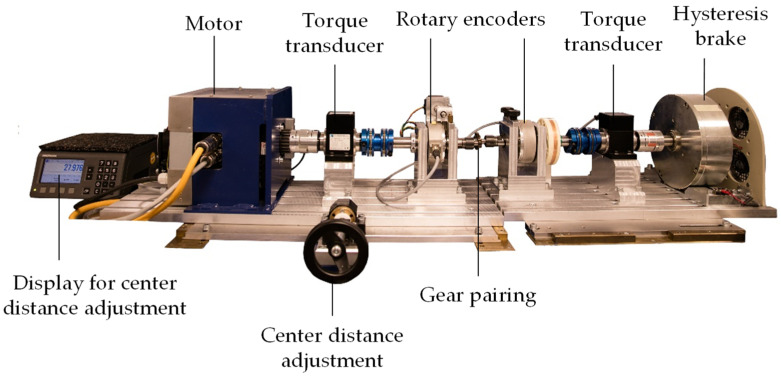
Main components of the in situ gear test rig of the LKT.

In consideration of the limitations of existing in situ gear test rigs, a new design has been developed at the LKT. The setup of the LKT in situ gear test rig is shown in Figure 1. The main components are a three-phase a.c. motor, type DSM150N by Baumüller, Nuremberg, Germany on the input side, and a hysteresis brake, type CHB-12 by Magtrol, Rossens, Switzerland on the output side.

**Figure 2:**
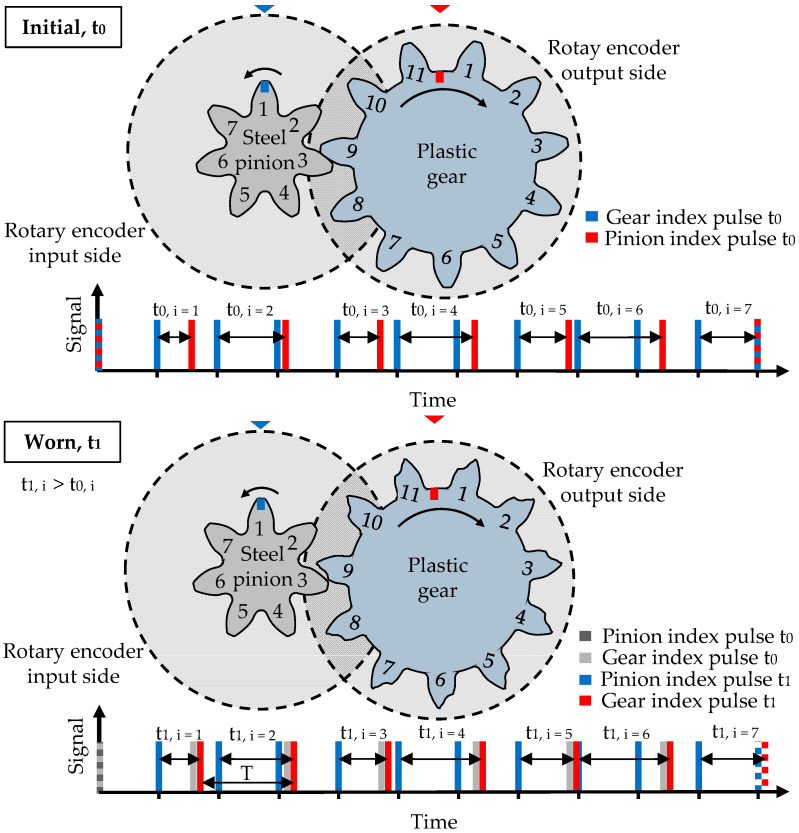
Tooth wear induced change of the timing difference between input and output index pulses, reprinted from Ref. [22].

The motor drives the steel pinion and the hysteresis brake generates the loading torque on the plastic gear. Torque fluctuations during the test run and the according frequency spectrum on the input and output side are measured by torque transducers, type TMB307 by Magtrol, Rossens, Switzerland. The tooth temperature of the plastic gear is measured continuously by positioning a thermocouple, type K, in a drilled hole with a diameter of 0.6 mm in the tooth root area, in the middle of the tooth. The temperature information is conveyed to a data logging PC via telemetry, type TEL1-PLM-IND by Kraus Messtechnik GmbH, Otterfing, Germany. The main measuring components for the tooth wear and deformation measurement are rotary encoders, type A020 by Fritz Kübler GmbH, Villingen-Schwenningen, Germany on the input and output shaft. Figure 2 shows the main components of the in situ gear test rig of the LKT.

In order to measure the tooth flank wear and elastic tooth deflection of the plastic gear on the output side, the index pulses of the rotary encoders on the input and output shaft are evaluated. With progressing wear and deformation, the index pulse of the gear delays. Thus, the timing difference between the input and output index pulses increases (t_0_ < t_1_) as shown in Figure 2.

With the time for one rotation of the plastic gear T on the output shaft, the angular displacement Δφ between pinion and gear can be calculated as follows:

(3) $$\Delta \varphi =\frac{∆t}{T}\cdot 360{}^{\circ}$$

The tooth flank wear and deformation Δs can be calculated with the angular displacement Δφ using Equation (4), at the measuring diameter d_Mk_ = 38.146 mm according to DIN 3977 [23]:

(4) $$\Delta s=\Delta \varphi \frac{\pi \cdot d}{360{}^{\circ}}$$

**Figure 3:**
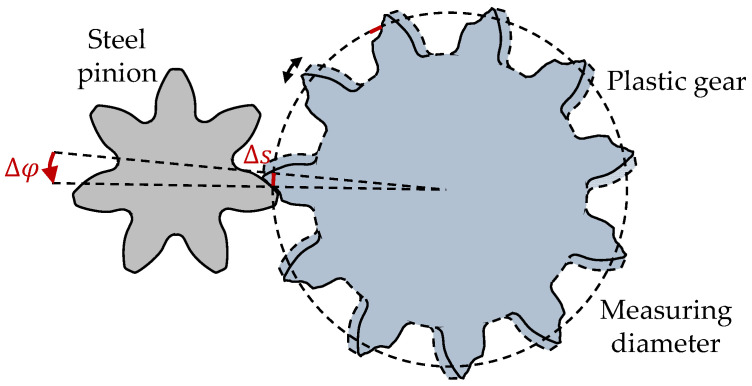
Wear and deformation induced angular displacement of pinion and gear, reprinted from Ref. [22].

The change of the timing difference Δt = t_1_ − t_0_ as an expression of the angular displacement between steel pinion and plastic gear is illustrated in Figure 3.

By switching between a high loading torque and a low measuring torque, the elastic component of the total deformation is removed. Thus, the remaining deformation (plastic deformation and wear), can be evaluated separate from the elastic deformation. One load cycle is defined as the time period until every tooth of the pinion and the gear has been in contact [24].

The resolution of the system depends on the rotational speed. At an input speed of 1000 rpm, the plastic gear rotates at approximately 435.9 min^−1^ and thus needs 0.138 s for 360° (one rotation). At the given sampling rate of 80 MHz, the encoder is able to sample every 0.0000000125 s which equals to 0.00003° rotation. This results in a tooth deformation measurement of ±0.01 µm assuming the plastic gear’s diameter of 39 mm. Therefore, the concept of evaluating timing differences has a high resolution even at higher speeds and therefore is suitable for testing during gear operation. Other concepts using rotary encoders measuring directly the angle on the contrary do not have a resolution which is high enough.

The functional principle of the in situ gear test rig has already been validated in terms of the measurement of the tooth flank wear and plastic deformation [25]. Nevertheless, a validation of the testing concept as far as the elastic tooth deflection measurement is concerned still needs to be done.

Within this work, the new measurement principle is validated regarding the measurement of the elastic tooth deflection by comparing the results of the test rig with those of pulsator tests. The new in situ measurement principle aims for a more comprehensive description of the operating behavior of plastic gears. By measuring the elastic deformations during operation, the influence of manufacturing-process-induced geometrical and morphological deviations of the injection molded plastic gears on the operating characteristics and lifetime can be analyzed in detail.

## 2. Materials and Methods

### 2.1. Materials and Specimens

For all gear tests, a wire cut steel pinion made of 100Cr6 was used. The plastic gears were made of PBT Ultradur B4520 by BASF AG, Ludwigshafen, Germany, which is a typical thermoplastic material used for gear applications. In addition, steel gears made of hardened 16MnCr5 were tested in order to evaluate the offset of the experimental setup. The technical specifications of the investigated pinion and gears according to DIN 867 [26] are shown in Table 1.

The plastic gears were injection-molded according to the processing data sheet [27] using an injection molding machine by Arburg GmbH Co. KG, Loßburg, Germany, type 370U-700-30-30. The main processing parameters are listed below (Table 2). The material was dried at 100 °C for 4 h before injection molding.

### 2.2. Testing Methods

To validate the elastic tooth deflection measurement, the results of in situ gear tests were compared to the results of pulsator tests. The correlation of the results was analyzed to evaluate the applicability of the newly developed testing concept for the measurement of elastic tooth deflections.

#### 2.2.1. Pulsator Tests

**Figure 4:**
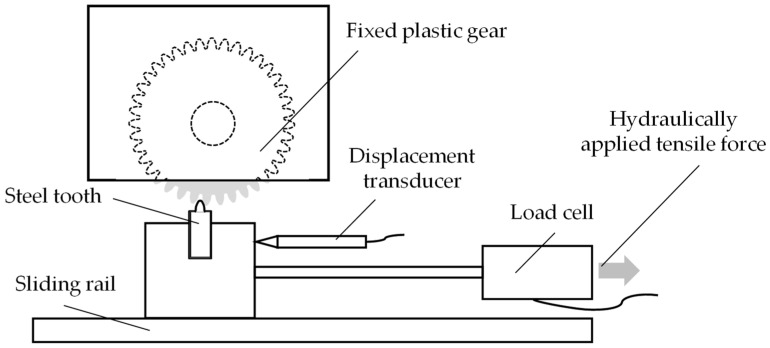
Pulsator test rig for the measurement of the elastic tooth deformation.

The used pulsator test rig by LUVRA Hydraulik und Regeltechnik GmbH, Nuremberg, Germany, consists of a steel tooth which is mounted on a sliding rail. The steel tooth is hydraulically pressed against a fixed polymer tooth. The horizontal movement of the plastic gear’s tooth due to the hydraulic force is measured by a displacement transducer. The pulsator test rig with its main components is shown schematically in Figure 4.

The plastic tooth was loaded sinusoidally and cyclically to evaluate the elastic tooth deformation. The frequencies and loads were chosen proportionately to the rotational speeds and loading torques of the in situ gear test runs. Since high loading speeds increase the elastic modulus of polymers [2], two different frequencies, 8.5 Hz (equals 510 rpm gear speed) and 16.7 Hz (1000 rpm gear speed), were tested. The loading force was varied between 26 N (0.50 Nm loading torque), 38 N (0.75 N loading torque), 51 N (1.00 Nm loading torque), 64 N (1.25 Nm loading torque), 77 N (1.50 Nm loading torque), 90 N (1.75 Nm loading torque) and 102 N (2.00 Nm loading torque). To prevent variations of the results because of temperature rises over time due to internal friction, the results for the tooth deflection were evaluated 10 s after the start of the test run. Since the test duration is only less than 1 s, the gear temperature was assumed to be constant at 23 °C. For the correlation of the gear test runs and the pulsator tests, only the first load cycle was evaluated since there is only a little fluctuation of the results over the short test duration.

#### 2.2.2. Gear Test Runs

**Figure 5:**
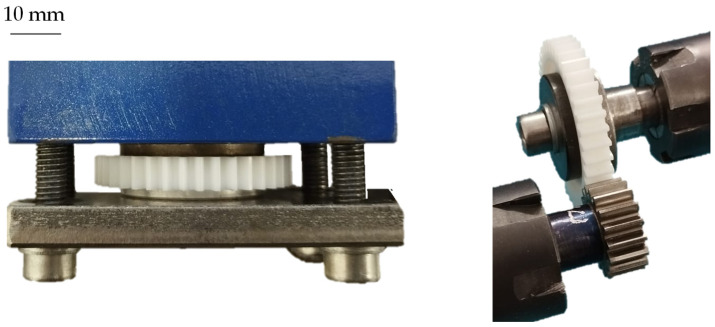
Clamping of the plastic gears, pulsator tests (**left**) and gear test runs (**right**).

To compare the gear test runs with the pulsator tests, the gear set was air-cooled during operation. Therefore, the tooth temperature measured via thermocouple in the tooth root was kept at a constant 23 °C. To ensure comparability between the two test procedures, the fixation of the gears was designed similarly. For this purpose, the plastic gears were clamped between two metal washers. Consequently, torsion of the plastic gear on the shaft, as it is prevalent when the plastic gear is directly molded onto the steel shaft, can be prevented. Figure 5 shows the clamping of the plastic gears used for the gear test runs and pulsator tests.

For the isothermal gear test runs, the high loading torque was applied for 200 load cycles and the low measuring torque for 40 load cycles. The test duration was set at three load changes. This results in a test duration of approximately 30 min at 510 rpm gear speed and 15 min at 1000 rpm gear speed. Different speed–load combinations have been investigated, which are typically relevant for gear applications. The low measuring torque was set at 0.2 Nm which is as low as possible without risking that the gears lose contact. The high loading torque was set at 0.50 Nm, 0.75 Nm, 1.00 Nm, 1.25 Nm, 1.50 Nm, 1.75 Nm and 2.00 Nm. Higher loads lead to early plastic deformations and gear failure and thus have not been investigated. The elastic tooth deflection was evaluated as the difference of the average deformation at the high torque and the average deformation at the low torque. The elastic tooth deflection only differs marginally between the first load change and the following two changes. Moreover, the plastic gear begins to wear during operation which would lead to incomparable test conditions. Consequently, only the first load change was evaluated in terms of the elastic tooth deflection for the correlation between the gear test rig and pulsator test rig.

## 3. Results and Discussion

### 3.1. Pulsator Tests

**Figure 6:**
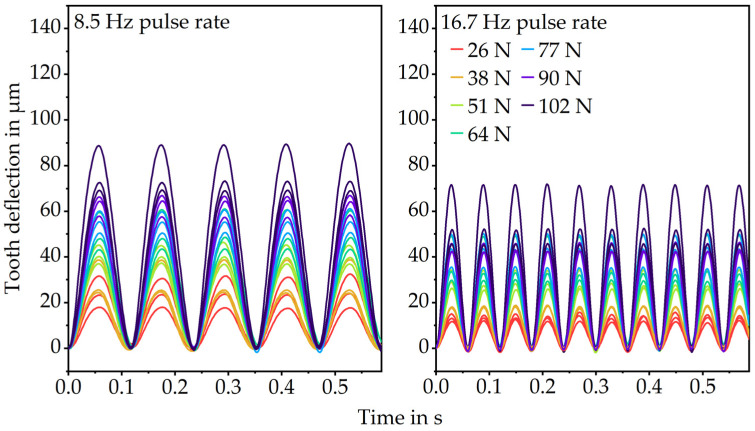
Results for the elastic tooth bending (pulsator tests).

As expected, the results of the pulsator tests show increasing elastic deformations with increasing loads, shown in Figure 6. The tooth deflection returns to zero when the load is removed for each load level. At the high pulse rate of 16.7 Hz, the deformations tend to be lower which is supported by the correlation between higher loading speeds and stiffer material behavior described in the literature [2].

### 3.2. Gear Test Runs

**Figure 7:**
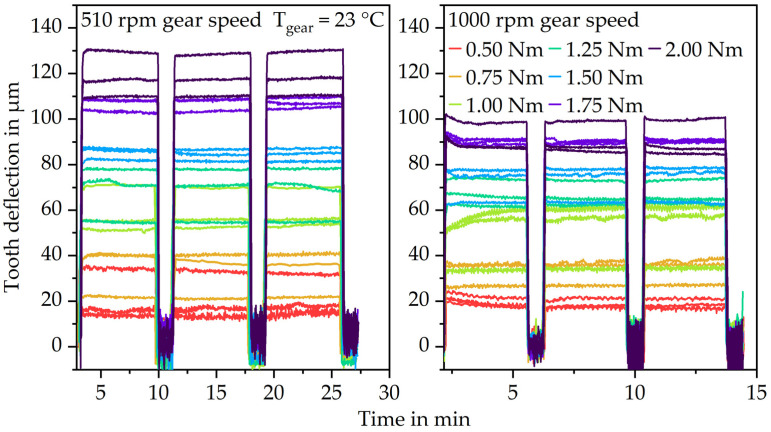
Results for the elastic tooth bending (gear test runs).

The results of the gear test runs (Figure 7) also show increasing elastic tooth deflection with increasing load. Furthermore, the elastic tooth deflection is lower at a 16.7 Hz pulse rate. Since the measured deformation at the low measuring torque fluctuates approximately ±10 µm around zero, the difference of the average deformation at the high torque and the average deformation at the low torque was evaluated as the elastic tooth deflection.

### 3.3. Correlation of Pulsator Tests and Gear Test Runs

**Figure 8:**
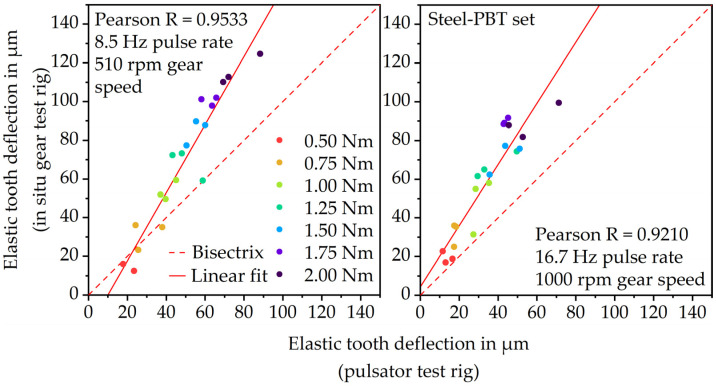
Correlation between the elastic tooth deflection measurement via in situ gear test rig and pulsator test rig.

Figure 8 shows the correlation between the elastic tooth deflection measured via the pulsator and in situ gear test rig at 8.5 Hz or 510 rpm (left) and 16.7 Hz or 1000 rpm (right). At 8.5 Hz and 16.7 Hz, the results show a linear increase in elastic deformation with increasing load. This indicates that both measurement principles are qualified for measuring the linear–elastic deformation behavior of plastic parts. At 8.5 Hz, the Pearson correlation coefficient of 0.9533 proves a very good correlation between the gear test rig and pulsator test rig results. At 16.7 Hz, the correlation coefficient 0.9210 is quite similar and also shows a very good correlation. However, at both frequencies, the values measured via the in situ gear test rig are higher than the values measured via the pulsator test rig. Due to the fact that during the gear test runs, a remaining measuring torque of 0.2 Nm is applied and possible load-sharing effects occur, the in situ measured elastic deformation is rather expected to be lower than the pulsator measured elastic deformation.

**Figure 9:**
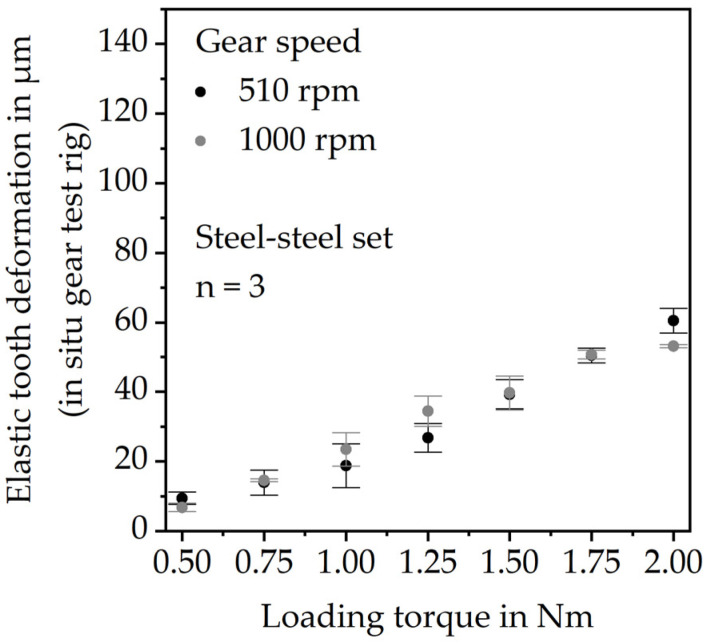
Measurement offset of the in situ gear test rig.

Thus, there is the suspicion that the test rig setup creates an offset measuring the elastic tooth deflection. This might be due to slight angular displacements of the components (e.g., shafts and fixations) within the setup during the test run. To verify this hypothesis and to quantify the offset, gear tests were performed using a steel gear instead of a PBT gear. Since the elastic modulus of steel is considerably higher, the investigated loads will not lead to tooth deflections. Hence, any deformation measured at the hardened 16MnCr5 gear is a measurement offset of the test rig setup. The gear tests with the steel-steel gear set were performed under grease lubrication. The steel gears were mounted within the experimental setup identically to the PBT gears. Figure 9 shows the results.

**Figure 10:**
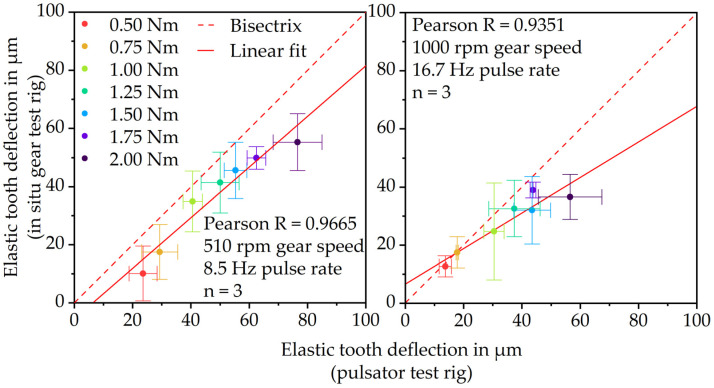
Offset adjusted correlation between in situ gear test rig and pulsator results for elastic tooth deflection.

To adjust the correlation, considering the offset of the test rig, the average deflection values of Figure 9 were subtracted from the average elastic tooth deflections at each load stage. The results are illustrated in Figure 10, also showing linearly increasing deformation with increasing load but at a much lower level. Taking the test rig offset into account, the elastic tooth deflection measured during operation via the in situ gear test rig still correlates very well with the deflection measured via the pulsator test rig. For both the 8.5 Hz and 16.7 Hz load frequency, the in situ gear test rig measures slightly lower deflections than the pulsator test rig, as expected. The lower in situ measured tooth deflection is primarily due to the low measurement torque of the in situ gear test rig which is still at 0.2 Nm. The hydraulically applied force of the pulsator test rig however is removed to zero cyclically. Further reasons might be the different kinematics. During the pulsator tests, the gear is loaded cyclically and horizontally at the pitch point. During the gear test run, however, there is a combination between sliding and rolling. Even though the force at the pitch point should be comparable, there still might be frictional losses resulting in lower deformations. Moreover, until now the load-sharing situation at different speed–torque combinations is not exactly clear, which might lead to lower tooth deflection in the actual gear test run.

## 4. Conclusions

The elastic tooth deflection of plastic gears influences the stress distribution of plastic gears and thus should be considered when designing plastic gear sets. However, no experimental approaches for the detection of elastic tooth deflections during operation exist in the literature.

Hence, this work presents a new test rig design which enables the understanding of the elastic tooth bending behavior of plastic gears during operation. Especially the influence of manufacturing parameters and resulting geometrical and morphological deviations of plastic gears on the operating behavior and the resulting lifetime can be analyzed in detail, using the new testing principle. The methodology has been validated by comparing the results to those of pulsator tests. The comparison shows very good correlations between gear test runs and pulsator tests for varying loads and speeds. The results show that the actual tooth deflection during operation is slightly lower than the deflection measured via the pulsator test rig. One reason for this is that the gear test rig measures at a low loading torque of 0.2 Nm, while the force of the pulsator test rig is reduced to zero cyclically. Further possible reasons are the sliding and rolling kinematics as well as load sharing during operation. By performing gear tests with a steel–steel pairing, it has been shown that the test rig creates an offset which increases linearly with the loading torque due to angular displacements of the test rig components.

Current and future research at the LKT focuses on the time-dependent interactions between tooth flank wear and elastic tooth deflection, their connection with the manufacturing process and their influence on the operational behavior and lifetime of plastic gears.

## Figures and Tables

**Table 1:** Technical specifications of the investigated gear sets.

DIN 867	Steel Pinion	Plastic Gear	Steel Gear
Picture	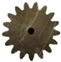	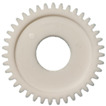	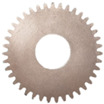
Material	Hardened 100Cr6	PBT Ultradur B4520	Hardened 16MnCr5
Module	1 mm		
Pressure angle	20°		
Number of teeth	17	39	
Gear width	8 mm	6 mm	
Profile shift	0.2045 mm	−0.3135 mm	

**Table 2:** Main processing parameters for the manufacturing of the plastic gears.

Processing Parameter	Parameter Setting
Screw diameter	18 mm
Mass temperature	260 °C
Mold temperature	60 °C
Injection/Holding/Cooling/Cycle time	2.2 s/6 s/25 s/42.8 s
Holding pressure	600 bar
Cylinder temperature profile (Nozzle → indentation)	260 °C/250 °C/240 °C/230 °C/90 ° C

## Data Availability

Not applicable.
